# Optimal strategy to identify incidence of diagnostic of diabetes using administrative data

**DOI:** 10.1186/1471-2288-9-62

**Published:** 2009-08-28

**Authors:** Shabnam Asghari, Josiane Courteau, André C Carpentier, Alain Vanasse

**Affiliations:** 1 PRIMUS Group, Centre de recherche clinique Étienne-Le Bel, CHUS, Sherbrooke (QC), Canada; 2 Departments of Medicine, Faculty of Medicine and Health Sciences, Université de Sherbrooke, Sherbrooke (QC), Canada; 3 Department of Family Medicine, Faculty of Medicine and Health Sciences, Université de Sherbrooke, 3001, 12th Avenue North, Sherbrooke (QC), Canada

## Abstract

**Background:**

Accurate estimates of incidence and prevalence of the disease is a vital step toward appropriate interventions for chronic disease like diabetes. A growing body of scientific literature is now available on producing accurate information from administrative data. Advantages of use of administrative data to determine disease incidence include feasibility, accessibility and low cost, but straightforward use of administrative data can produce biased information on incident cases of chronic disease like diabetes. The present study aimed to compare criteria for the selection of diabetes incident cases in a medical administrative database.

**Methods**

An exhaustive retrospective cohort of diabetes cases was constructed for 2002 using the Canadian National Diabetes Surveillance System case definition (one hospitalization or two physician claims with a diagnosis of diabetes over a 2-year period) with the Quebec health service database. To identify previous occurrence of diabetes in the database, a five-year observation period was evaluated using retrograde survival function and kappa agreement. The use of NDSS case definition to identify incident cases was compared to a single occurrence of an ICD-9 code 250 in the records using the McNemar test.

**Results**

Retrograde survival function showed that the probability of being a true incident case after a 5-year diabetes-free observation period was almost constant and near 0.14. Agreement between 10 years (maximum period) and 5 years and more diabetes-free observation periods were excellent (kappa > 0.9). Respectively 41,261 and 37,473 incident cases were identified using a 5-year diabetes-free observation period with NDSS definition and using a single ICD-9 code 250.

**Conclusion**

A 5-year diabetes-free observation period was a conservative time to identify incident cases in an administrative database using one ICD-9 code 250 record.

## Background

Diabetes is one of the most costly and burdensome chronic diseases of our millennium [1]. According to the latest World Health Organization report, more than 180 million people worldwide suffer from diabetes. This number will very likely double by 2030[2]. Therefore, public health prevention and intervention are urgently needed.

Planning, implementing and monitoring of appropriate intervention for this disease requires accurate estimates of incidence and prevalence of the disease. Although surveys are used for precise estimation of prevalence and incidence, they are very expensive and time consuming. Alternative methods include secondary analysis of existing data and use of administrative data [3,4]. Advantages of use of administrative data to estimate disease incidence include easy access, low cost, and availability of data over long and continuous periods. Research by Blanchard and colleagues in Manitoba and Hebert et al. in the United States showed that health care administrative data can be used to identify individuals diagnosed with diabetes mellitus and estimate rates over time[1,5,6]. Occurrence of diabetes was defined as two physician claims with a diagnosis of diabetes (ICD-9 code 250) on 2 different days within 2 years or one hospital discharge with a diabetes diagnostic code in any of the 16 diagnosis fields. They found an approximately 95% sensitivity of their diabetes case definition from administrative data compared to a diabetes education registry [1,6]. As a result, this diabetes case definition algorithm has been adopted by the Canadian National Diabetes Surveillance System (NDSS) [7].

However, there are several potential pitfalls of administrative data to estimate disease incidence [8]. One general problem is representativeness of administrative data. In Canada with universal health insurance coverage, the health services are provided to all residents; therefore, almost all Canadians are represented by these datasets (details available from the Canada Health Care System website) [9]. The other challenge in this respect is related to multiple utilizations of health services by a single patient. In principle, record linkage allows detection of multiple records of the same patient [8,10], but it is difficult to define which records should be considered to be the starting point of the care episode. In this paper, we compare different methods to identify incident cases of diabetes using Quebec health service data.

## Methods

### Study Database

A retrospective population-based cohort was constructed using data from the *Régie de l'Assurance Maladie du Québec *(RAMQ) and the registry *Maintenance et exploitation des données pour l'étude de la clientèle hospitalière *(Med-Echo). The RAMQ provides universal health insurance for Quebec inhabitants, which includes coverage for physician and hospital services, as in other Canadian provinces. For each physician service, the patient's identification, date of service, diagnosis (a four digit ICD-9 code), and service code are entered into a "physician claims" database. Similarly, after each hospital discharge, Quebec hospitals submit a discharge summary that includes the patient's identification, dates of admission and discharge, attending physicians, main reason for admission and up to 16 ICD-9 diagnoses. These hospital discharge records constitute the Med-Echo registry. The accuracy of this administrative health data has been demonstrated for a number of medical conditions [11,12]. All databases were also linked using a unique encrypted number that preserved the exact identification of individuals but allowed for the examination of individuals across the administrative databases.

### Study Population

The study population includes all individuals 20 years old or older living in the province of Quebec in 2002. The date of the first claim for diabetes or hospitalization in the year 2002 was defined as the index date. We excluded women with gestational diabetes mellitus. To eliminate gestational diabetes any record with an obstetrics event within 5 months of the index date was excluded.

### Methodology

A single cohort was selected to determine incident cases of diabetes. The 2002 cohort was selected as an example of application. This cohort provided 10 years of retrospective observation to exclude previous cases. A diabetes case was defined according to the Canadian National Diabetes Surveillance System (NDSS) case definition: two physician claims with a diagnosis of diabetes (ICD-9 Code 250) on 2 different days within 2 years or one hospital discharge with a diabetes diagnosis code in any field among 16 diagnosis codes. To find the cases we started from the date of the first claim for diabetes or hospitalization in the year 2002, whichever came first. To apply the definition, the databases were observed for the 720 days before the index date. To differentiate between prevalent cases and incident cases, a minimum diabetes-free retrospective observation period (clearance period) and a definition for repeated cases were needed. Our options for identifying repeated cases were using NDSS case definition (one hospitalization record or two physician claims during 2 years) or using a single record of ICD-9 code 250 (in hospital database or medical service database) during the retrospective observation period. In this study NDSS case definition criteria is called "NDSS method" and a single record of ICD-9 code 250 is called "one hit method ". To define the cut-off point for a clearance period for each index case in 2002, the backward time to the previous event was separately calculated using NDSS method and using the one hit method. Time was measured in days from the index point. The maximum (backward) observation period was 3650 days.

**Figure 1:**
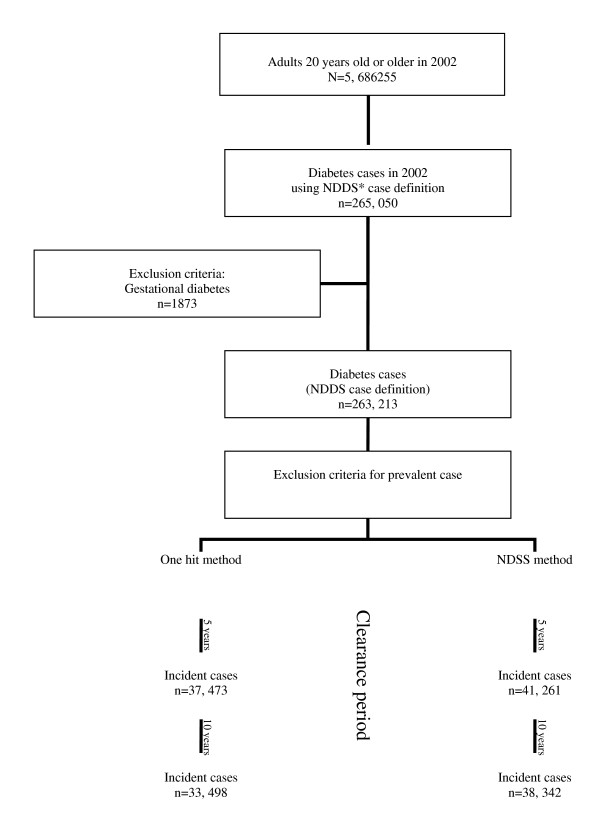
**Selection criteria to identify incident cases of diabetes in the Quebec health service database**. *NDSS: National Diabetes Surveillance System case definition in Canada

The Population at risk of diabetes at the beginning of the year 2002 includes people not diagnosed with diabetes and new cases of diabetes. As it was shown in figure 1, the population not diagnosed with diabetes before 2002 was determined (population at risk at the end of the year 2002). Then the population with a diagnosis of diabetes before 2002 (prevalent cases) was also determined. The newly diagnosed cases in the year 2002 were identified representing incident cases of diabetes during 2002.

### Statistical Analysis

Descriptive statistics and McNemar matched pair chi-square test are used to compare the NDSS method and the one hit method to exclude prevalent cases.

Kappa statistics is used to show the level of agreement between different lengths of observation periods and ten years as the maximum observation period. Kappa values are categorized as follows by Byrt[13,14] values between -1 and 0 indicate "no agreement"; between 0 and 0.20 indicate "poor agreement"; between 0.21 and 0.40 indicates "slight agreement"; between 0.41 and 0.60 indicates "fair agreement"; between 0.61 and 0.80 indicates "good agreement"; between 0.81 and 0.90 indicates "very good agreement"; and between 0.91 and 1.0 indicates "excellent agreement".

Next, The probability of being an incident case (being free of retrospective event) at different times (cut-points) is calculated by retrograde survival function. By this method, survival (being an incident case) at a given time is the conditional probability of surviving (not having a preceding record) to a specific time given that the individual is at risk for the event (having a preceding record) at that time. To find the cut-off point, we assumed that there is a time when all previous cases have been accounted and the patient is no longer at risk of a previous event. The cut-off point of the clearance period will be considered the point that the probability for being at risk of a previous event begins to remain constant. Statistical analyses were performed using SAS 9.1[15].

### Ethical considerations

This project was approved by the Ethics Review Board of the *Faculté de médecine *of the *Université de Sherbrooke *and the *Commission d'accès à l'information du Québec*.

## Results

There were 5,686255 adults, 20 years old or older for the year 2002. A total of 263,213 patients met the NDSS case definition for being a diabetes case (figure 1). Using the one hit method provided 33,498 incident cases (incidence proportion 1.08) over a clearance period of ten years. Applying the NDSS method and a ten-year clearance period, 38,342 incident cases of diabetes (incidence proportion 1.24) were found. There was a significant difference between the two case definition criteria using the McNemar matched pair chi-square test (*P *< 0.0001).

Cross-tabulations of incident cases and duration of the clearance period shows that diabetes incident cases decrease when the clearance period increases (Table 1).

**Table 1:** New cases of diabetes and kappa agreement by clearance period and exclusion criteria

	Exclusion criteria for prevalent case					
	
	One hit method			NDSS* method		
**Clearance period (years)**	Number of new cases	Incidence proportion**	Kappa agreement***	Number of new cases	Incidence proportion**	Kappa agreement***

1	91872	2.9%	0.427 (0.420-0.432)^†,§^	91872	2.9%	0.482 (0.479-0.485)^†,§^

2	52508	1.7%	0.739 (0.731-0.742)^†,§^	52508	1.7%	0.810 (0.801-0.822)^†,§^

3	43473	1.4%	0.849 (0.845-0.851)^†,§^	45525	1.5%	0.898 (0.896-0.900)^†,§^

4	39595	1.33%	0.900 (0.900-0905)^†,§^	42791	1.4%	0.936 (0.933-0.937)^†,§^

5	37473	1.23%	0.935 (0.933-0.937)^†,§^	41261	1.3%	0.957 (0.956-0.958)^†,§^

6	36111	1.16%	0.957 (0.955-0.958)^†,§^	40218	1.29%	0.971 (0.970-0.973)^†,§^

7	35094	1.14%	0.973 (0.972-0.974)^†,§^	39475	1.28%	0.982 (0.981-0.983)^†,§^

8	34400	1.11%	0.985 (0.983-0.986)^†,§^	38975	1.26%	0.990 (0.989-0.991)^†,§^

9	33932	1.09%	0.993 (0.992-0.994)^†,§^	38627	1.25%	0.996 (0.995-0.996)^†,§^

10	33498	1.08%	100	38342	1.24%	100

*: National Diabetes Surveillance System case definition in Canada.

**: Incidence proportion = (Number of incident cases of diabetes for the year 2002/population at risk of diabetes for the year 2002)*100; number of diabetes cases for the year 2002 using NDSS case definition criteria is 263,213; population of 20 years old or older for the year 2002 is 5,686,255;

***: Agreement between 10 years (expected) and observed clearance period according to the clearance period and exclusion criteria for prevalent case;

^†^: 95%Confidence interval;

^§^: *P *< 0.0001, Chi-square test statistic for homogeneity.

Using the one hit method, the agreement between a ten-year and a nine-year clearance period is excellent (kappa > 0.99). The agreement is also excellent between a ten-year clearance period and clearance periods of four years or more (kappa > 0.90). The agreement changed to very good (kappa > 0.85), good (kappa > 0.74), and fair (kappa > 0.43) when a three, two and one-year clearance period was used, respectively. Results using the NDSS method were similar and showed that agreements between a ten-year clearance period and clearance periods of four years and more were excellent (Table 1).

**Figure 2:**
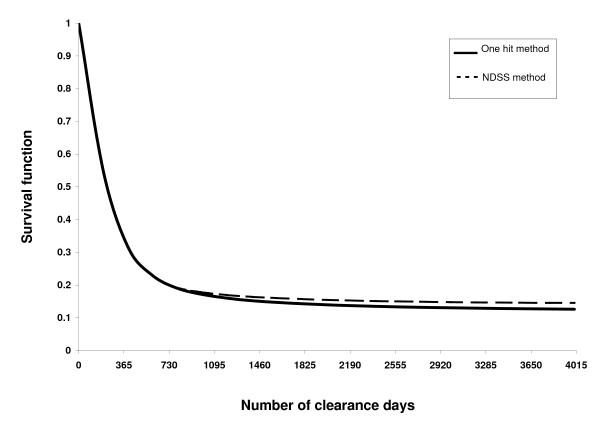
**Retrograde survival curve showing the proportion of new cases by exclusion criteria and clearance days**. *NDSS: National Diabetes Surveillance System case definition in Canada

The estimation using survival function by one hit method for eliminating prevalent cases at one year (365 days) was (0.35, SE = 0.0009) (Figure 2). There was a reduced and non-constant risk for a preceding record of diabetes occurring at 1600 days (0.15, SE = 0.0006) before the index point in 2002 and the observed risk remained constant 1900 days prior to the index point (0.14, SE = 0.0006). We observed a similar pattern using the NDSS method, i.e. the observed risk remained constant 1900 days prior to the index point (0.15, SE = 0.0006).

## Discussion

Previous studies on diabetes incidence for Canadian adults from administrative data relied on the NDSS (National Diabetes Surveillance System) case definition and employed a free observation period to remove the prevalent pool effect [1,6]. In our study, the retrograde survival function showed that a five-year clearance period is a reliable clearance period to distinguish new cases in a prevalent pool. Even if the kappa agreement is excellent after four years we did proceed with retrograde survival function to take into account the high prevalence of patients with diabetes in the database.

Diabetes is an insidious disease which may go undiagnosed for many years. In this retrograde method, we started with the diabetes cases who already met the criteria of NDSS in the year 2002. Canadian studies show the NDSS case definition has a sensitivity above 86%; specificity around 97%; and positive predictive values of 80% [1,6]. We aimed to eliminate prevalent cases from incident cases identified by the NDSS case definition so that high sensitivity and less selection bias were of main interest. According to our study, there was a significant difference between the NDSS method and the one hit method (P < 0.0001) to identify diabetes incident cases. The difference is 1.4% (3788) for a 5-year clearance period using one hit method and 1.8% (4844) for a 10-year clearance period for the NDSS method. This finding confirms those of previous studies [6]. As reported by Wilson, the use of more than one ICD-9 code 250 to find diabetes cases in an administrative database for a three- year period results in significant loss of sensitivity, and specificity of a single ICD-9 code 250 is nearly the same as the use of two ICD-9 codes 250 [16]. On the other hand this result could be interpreted within the context of the study's design. A large administrative dataset, provides sufficient sample size to observe relations, and the more restricted definition for NDSS method (i.e. Two physician visits or one hospitalization within two years) than the one hit method thus for choosing between two methods, considering both work similarly, an easier method which is slightly more sensitive is superior to a more difficult method which is somewhat less sensitive.

In practice, it is usual to exclude patients who received the same diagnosis several years before the beginning of the study period [6,1,17]. Previous studies used different cut-off points for clearance periods based on the length of registration years in the databanks [6,1,17]. In our study, we used the kappa agreement method [13] and retrograde survival function to validate the optimal clearance period for determination of diabetes incidence from administrative data.

We found excellent agreement (kappa > 0.90) between a 10-year clearance period and a clearance period of 4 years or more. However such a high agreement (kappa > 0.90) may be related to the high prevalence of cases in our database [13]. For this reason, we continued our analysis by retrograde survival function. This method showed that five years is an appropriate duration for a clearance period and the observed risk of being a repeated case remained constant thereafter.

The accuracy of our incidence estimation may also be affected by validity of diagnosis codes recorded in administrative databases and time. Canadian studies on administrative data showed that there is a good agreement between recorded cases in administrative databases and self-reported disease [11,18]. To evaluate the effect of the cohort of index time (2002 in our study) on the chance of being a preceding case, we evaluated reproducibility of a 10-year vs. 5-year clearance period for different index years including 1999, 2000. We found kappa > 0.90 in all cases (data not shown).

We also evaluated the consistency of a five-year clearance period when different selection criteria were used. Kappa were > 0.90 between a clearance period of 10 years vs. a clearance period of 4 years or more whether one hit method or NDSS method were used.

Our study has some limitations. We used medico-administrative data to find the incident cases. This may underestimate the real incidence because most people with diabetes remain asymptomatic for years after onset of the disease and because only patients who received health care services are entered in these databases [19,20]. In Canada with universal insurance coverage, every encounter with the health system is recorded in medico-administrative registries. To ensure patients encountered the health services during the clearance period (of any diagnosis) we examined the 37473 incident cases of diabetes for records related to other services and only 1301 (3.5%) had no record related to another kind of service. There are three possible conditions that could result in non-detection of potential cases of diabetes during the study period:

1-People who did not need the services; 2-diabetic patients who used services for other reasons; 3-sick people who left the country.

One study in Manitoba showed the probability of 0.96 for diabetic patients to have a subsequent medical contact for the diabetes within two years [1]. A validation study using patient records and administrative data in Ontario showed sensitivity for diabetes was 90 and 86%; for the 1- and 2-claim algorithms, specificity was 92 and 97%, respectively, and positive predictive values were 61 and 80%, negative predictive values were 99 and 98%, respectively [6]. On the basis of Canadian census data on out-of-country and out-of-province migrations, we estimated that fewer than 5% of these patients would have migrated in or out of Quebec during the study period (details available from Statistics Canada). Therefore the missed data was unlikely to substantially affect the reported results.

In our database, it was not possible to differentiate between diabetes type 1 and type 2.

The results may not be representative for the younger populations with diabetes because patterns and temporal evolution of disease is different between diabetes type 1 and type 2 and incidence of the former is higher in young populations. Furthermore, we did not assess clearance periods of more than ten years. However the results of our retrograde survival function analyses showed stabilization of risk of being a previous case after a clearance period of 5 or more years.

## Conclusion

Use of recorded administrative data to estimate incidence of diabetes using either the Canadian National Diabetes Surveillance System definition or one ICD-9 code 250 produces biased estimates in part because of inclusion of prevalent cases as incident cases. A clearance period of 5 years or more is sufficient to improve performance of this convenient, accessible and inexpensive method to estimate incidence of diabetes.

## Competing interests

ACC regularly consults for many pharmaceutical companies on lipid and diabetes drugs. He is also involved in many research contracts with the pharmaceutical industry. Moreover, he received funding from GSK to create a CIHR/industry chair on diabetes.

Neither the funding sources nor author contracts played a role in the study's design, conduct, or reporting or in the decision to submit the article for publication.

## Authors' contributions

All authors participated in the study's conception and design. AV, SA, and JC developed the methodology and analysis approach. SA drafted the manuscript. AV and JC helped draft the manuscript. All authors read and approved the final manuscript.

## Pre-publication history

The pre-publication history for this paper can be accessed here:

http://www.biomedcentral.com/1471-2288/9/62/prepub

## References

[B1] Blanchard JF Ludwig S Wajda A Dean H Anderson K Kendall O Depew N Incidence and prevalence of diabetes in Manitoba, 1986-1991 Diabetes Care 1996 19 8 807 811 10.2337/diacare.19.8.807 8842595

[B2] Diabetes facts- fact sheet N°312, 2006 2006 World Health Organization, Geneva, Switzerland http://www.who.int/mediacentre/factsheets/fs312/en/index.html Accessed September, 1, 2008

[B3] Valdes S Botas P Delgado E Alvarez F Cadorniga FD Population-based incidence of type 2 diabetes in northern Spain: the Asturias Study Diabetes Care 2007 30 9 2258 2263 10.2337/dc06-2461 17536076

[B4] Brocco S Visentin C Fedeli U Schievano E Avogaro A Andretta M Avossa F Spolaore P Monitoring the occurrence of diabetes mellitus and its major complications: The combined use of different administrative databases Cardiovascular Diabetology 2007 6 5 doi:10.1186/1475-2840-6-5 10.1186/1475-2840-6-5 17302977 PMC1804263

[B5] Hebert PL Geiss LS Tierney EF Engelgau MM Yawn BP McBean AM Identifying persons with diabetes using Medicare claims data Am J Med Qual 1999 14 6 270 277 10.1177/106286069901400607 10624032

[B6] Hux JE Ivis F Flintoft V Bica A Diabetes in Ontario: determination of prevalence and incidence using a validated administrative data algorithm Diabetes Care 2002 25 3 512 516 10.2337/diacare.25.3.512 11874939

[B7] Diabetes in Canada 2002 Center for chronic disease prevention and Control, Population and Public Health Branch, Health Canada 2003 Ottawa, Ontario, Canada: Public Health Agency of Canada http://www.phac-aspc.gc.ca/publicat/dic-dac2/english/01cover-eng.php Accessed September, 1, 2008

[B8] Sorensen HT Sabroe S Olsen J A framework for evaluation of secondary data sources for epidemiological research International Journal of Epidemiology 1996 25 2 435 442 10.1093/ije/25.2.435 9119571

[B9] Richards J Brown A Homan C The Data Quality Study Of The Canadian Discharge Abstract Database Proceedings of Statistics Canada Symposium 2001. Achieving Data Quality in a Statistical Agency: A Methodological Perspective http://www.statcan.gc.ca/bsolc/olc-cel/olc-cel?catno=11-522-X20010016282&lang=eng Accessed June, 1, 2009

[B10] Sund R Utilization of routinely collected administrative data in monitoring the incidence of aging dependent hip fracture Epidemiologic Perspectives and Innovations 2007 4 2 doi: 10.1186/1742-5573-4-2 10.1186/1742-5573-4-2 17555560 PMC1899499

[B11] Palin JA Zumbo BD Measurement of health care utilization in Canada: Agreement between surveys and administrative records 2003

[B12] Tamblyn R Lavoie G Petrella L Monette J The use of prescription claims databases in pharmacoepidemiological research: the accuracy and comprehensiveness of the prescription claims database in Quebec J Clin Epidemiol 1995 48 8 999 1009 10.1016/0895-4356(94)00234-H 7775999

[B13] Szklo M Nieto FJ Epidemiology: Beyond the Basics 2007 2 Jones and Bartlett Publishers

[B14] Byrt T Bishop J Carlin JB Bias, prevalence and kappa J Clin Epidemiol 1993 46 5 423 429 10.1016/0895-4356(93)90018-V 8501467

[B15] SAS Institute Inc SAS Version 9.1 2003

[B16] Wilson C Susan L Lynch A Saria R Peterson D Patients with diagnosed diabetes mellitus can be accurately identified in an Indian Health Service patient registration database Public Health Rep 2001 116 1 45 50 11571407 10.1016/S0033-3549(04)50021-3 PMC1497292

[B17] Lipscombe LL Hux JE Trends in diabetes prevalence, incidence, and mortality in Ontario, Canada 1995-2005: a population-based study Lancet 2007 369 9563 750 756 10.1016/S0140-6736(07)60361-4 17336651

[B18] Shah BR Hux JE Laupacis A Zinman B Cauch-Dudek K Booth GL Administrative data algorithms can describe ambulatory physician utilization Health Serv Res 2007 42 4 1783 1796 10.1111/j.1475-6773.2006.00681.x 17610448 PMC1955277

[B19] McGlynn EA Health information systems: design issues and analytic applications 1998 Santa Monica, CA: Rand

[B20] Ekoé Jean Marie Zimmet Paul Williams Rhys The Epidemiology of Diabetes Mellitus: An International Perspective 2001 Wiley, Chichester

